# Replies to the Critics of Knowing and Checking: an Epistemological Investigation

**DOI:** 10.1007/s12136-022-00541-0

**Published:** 2023-01-04

**Authors:** Guido Melchior

**Affiliations:** Department of Philosophy, University of Graz, Heinrichstrasse 26/5, 8010 Graz, Austria

## Abstract

This paper replies to the comments made in *Acta Analytica* by Peter Baumann, Kelly Becker, Marian David, Nenad Miščević, Wes Siscoe, and Danilo Šuster on my *Knowing and Checking: An Epistemological Investigation* (Routledge 2019), hereinafter abbreviated as KC. These papers resulted from a workshop organized by the department of philosophy of the University of Maribor. I am very thankful to the organizers of the workshop and to the authors for their comments.

## Peter Baumann: Checking Out Checking

I am indebted to Peter Baumann for his comments, who is one of the most informed and distinguished epistemologists I know. He raises four objections against KC: First, he questions the modal profile and the externalist nature of checking. Second, he suggests, against KC, that there is only contrastive checking and no checking simpliciter. Third, he criticizes the view endorsed in KC that checking is not closed under known entailment. Finally, he makes two smaller points: first, concerning my claim that checking does not suffer from the generality problem, and second, he proposes a further version of the heterogeneity problem as introduced in Melchior (2015). I will reflect stepwise on each of these objections.

### The Modal Profile and the Externalist Nature of Checking

As a first point, Baumann addresses the question of whether the modal sensitivity account of KC matches our natural understanding of the verb “checking.” As Baumann (forthcoming) notes: “There is an ordinary concept of checking but one can also construct more or less technical notions of checking. In the following I will raise some questions and doubts about whether Guido’s notion of checking fits with our ordinary notion.” Baumann raises doubts about whether the notion of checking developed in KC really matches our natural understanding, thereby suggesting that it could be a more technical notion. He presents cases of subjects who intentionally use methods for checking, ordinary inductive reasoning in one case and deductive reasoning in another case, which happen to be insensitive. Baumann argues that given that the methods delivered the correct result, we tend to judge the subjects checked, despite of the insensitive methods used.

I do not claim in KC that there is a single correct use of “checking.” As I discuss, “checking” can refer to the intentions and actions of the checking subject or to an act of successful checking that delivers a certain (correct) result. In KC, I am interested in checking in the last sense. Baumann’s cases of insensitive (and unsafe) checking seem to fall under the first category. I do not say much in KC about the cases that Baumann brings up, but I agree with him that there is an intuitive pull towards judging that a subject checked if she intentionally used a method that delivered the correct result given that the method has some epistemic virtues, even if it lacks sensitivity. Importantly, Baumann presents cases of ex post judgments. However, our linguistic intuitions pull into the opposite direction in cases of ex ante reports. From an ex ante point of view, *before* the subject checks, it seems odd to say that she can successfully check by using a method with the wrong modal profile, e.g., with a monotonous method that always delivers the same result, regardless of whether *p* is true or false. In Baumann’s cases, I think we intuitively focus on and overemphasize the fact that the method delivered the correct result, ignoring how we came to the result. In contrast, from an ex ante point of view, before we know what the method will indicate, we can only focus on the method’s modal profile. In KC, I am interested in a particular strong understanding of checking that involves intentionally using an appropriate method. As I write in KC (33):

When talking about checking we usually also consider the appropriateness of the method used. This is the understanding of checking in which I am interested. For example, we want to say that Mary does not check whether the brakes of her car work properly if she tosses a coin for determining whether they do so. In this case, the method of checking is inappropriate. Thus, the way I use ‘checking whether* p* is true’ implies using an appropriate method with respect to *p*.

Baumann is right that this narrow understanding of checking might not be in line with all our uses of the verb “checking.” This points towards a certain ambiguity of the verb “checking.” Checking is a process and we can reflect about it from an ex ante point of view, while the subject is checking, and from an ex post point of view, when the process has delivered a certain result. Knowledge, in contrast, is a state preventing a similar distinction between ex ante and ex post points of view. Perhaps the concept of checking is, therefore, more ambiguous than other epistemic concepts such as knowing.

Baumann also argues, via examples, that there is plausibly a usage of “checking” that is not factive. For example, he argues that we can check by using Newtonian physics, although relativity theory is actually true. Admittedly, there is some plausibility to claiming that we can check by using Newtonian physics. However, this is a general problem within the philosophy of science and not a problem for checking in particular. Just like we can ask whether we can check using Newtonian physics, we can also raise the question whether Newtonian physics is in some sense *true* or whether we can *know* via Newtonian physics. Furthermore, Baumann presents the example of Frege who checks by going through some proof using his ill-fated fifth axiom as a case of non-factive checking. I do not find this example persuasive either. In this case, I am inclined to judge that Frege checked in the sense of intentionally using a method for settling a question, but he did not successfully check in the full-fledged sense I am interested. Finally, Baumann (forthcoming) presents the following case:

Assume that two chemists are noticing that a certain substance has caught fire under condition C. They wonder which substance it is. Chemist A looks up the relevant chart (published by the Chemistry Society) which says that only Phosphorus ignites under condition C. So far, no exception to this has been found. A then tells his colleague B: “It’s phosphorus!” B asks: “How do you know?” And A replies: “I just checked – I looked up the chart.” A has used a testimony-mediated inductive method. It is not weakly sensitive. The method would still indicate that it’s phosphorus even if that was false. But using such a method would still count as a case of checking in the ordinary sense.

In this case, I do not see why the method that Baumann sketches is insensitive as Baumann claims. Plausibly, catching fire under condition C is a necessary property of phosphorus (and that it is also necessary that no other substance ignites under condition C). In this case, the counterfactual “If phosphorus did not catch fire under condition C, then the chart would not indicate that it ignites under condition C” is a counterpossible (a counterfactual with an impossible antecedent). Necessities and counterpossibles are tricky terrain for modal knowledge accounts because they tend to be vacuously true which is often regarded as having implausible consequences. If we opt for standard possible world semantics, then counterpossibles are vacuously true and sensitivity is trivially fulfilled. If we opt for a non-standard impossible worlds account, as I sketch in KC and develop further in Melchior (2021b), then counterpossibles can turn out false, making the method used insensitive. However, in these cases, impossible worlds where phosphorus does not ignite under condition C and the chart mistakenly reports that it ignites under condition C are more remote than impossible worlds where phosphorus does not ignite under condition C and the chart correctly indicates that. Again, the method turns out to be sensitive, in contrast to Baumann’s diagnosis. Consulting a reliable chart can only be an insensitive inductive method, as Baumann claims, if the chart indicates (correctly) that phosphorus frequently but not necessarily ignites under condition C (or that other substances can also ignite under condition C) and the chemist infers from that claim that the substance is phosphorus. However, I would assume that in this case, we would no longer judge anymore that the chemist really checked.

### Contrastive Checking

The second point that Baumann addresses is the contrastive nature of checking. In KC, I draw a distinction between checking simpliciter whether *p* is true and contrastive checking whether *p* or a particular alternative *q* is true. These types of checking differ along two dimensions, the intentions of the checking subject and the sensitivity conditions that must be fulfilled. Checking simpliciter whether* p* is true involves the particular intention of checking whether* p* is true or false and requires fulfillment of the classical sensitivity condition, namely, that method M would not indicate that* p* is true if* p* were false. Contrastive checking, in contrast, involves the particular intention of checking whether* p* *or* *q* is true and fulfillment of the sensitivity condition that M would not indicate that* p* is true if *q* instead of *p* were true.

I hold in KC that checking simpliciter is a genuine form of checking. Baumann objects that checking simpliciter is reducible to contrastive checking, proposing “an argument for reduction, not for elimination of the concept of checking simpliciter.” Baumann presents subtle cases suggesting that there is no checking that Peter cleaned the kitchen without contrastive checking, e.g., that Peter and not someone else cleaned the kitchen. Baumann (forthcoming) concludes:

So, it seems we end up with a moderate reductive view of checking: Checking simpliciter requires and reduces to at least some contrastive checking. It is perhaps never required to do all the contrastive checking but always some. What and how much contrastive checking is required, varies with contexts of inquiry.

How can we evaluate Baumann’s proposal? First of all, let me note that checking simpliciter *is* a form of contrastive checking. Contrastive checking requires checking that* p* and not a particular alternative* q* is true. In case of checking simpliciter, the alternative that has to be ruled out is simply ~ *p*. In this sense, there is no categorical difference between checking simpliciter and contrastive checking, and Baumann is right that checking simpliciter requires and reduces to contrastive checking and that all checking is contrastive checking. Moreover, I am fine with Baumann’s cases where checking simpliciter entails a particular form of contrastive checking. I also think that in many cases (or even in most), the checking subjects might have the intention to check whether* p* and not a particular alternative* q* that is distinct from ~ *p* is true. It is in this context that the claim that “checking whether a proposition is true simpliciter … might be the exception.” (KC 96) must be understood, a claim that puzzles Baumann. However, I disagree with Baumann that checking simpliciter can *always* be reduced to some other form of contrastive checking. I think there are numerous forms of checking that do not involve any further intentions or interests of the checking subject concerning a particular alternative. Take the example of checking whether there is milk in the fridge. What we normally aim at in this case is just checking whether there is milk in the fridge or not; we do not think of or are interested in specific alternatives, e.g., whether there is milk or yoghurt in the fridge, or whether there is milk in the fridge and not in the oven. I do not see how these instances of checking can be plausibly reduced to checking of particular alternatives other than ~ *p*. For these cases, KC’s analysis of checking simpliciter as irreducible to other forms of contrastive checking seems correct, although contrastive checking might be fundamental in some other cases.

### Closure

Whether knowledge is closed under known entailment has been subject of a number of recent philosophical discussions. I argue in KC that checking is not closed under known entailment, leaving open whether knowledge is closed.^1^ Against this, Baumann argues that checking fulfills some version of the closure principle.

In KC, I defend a modal notion of checking, with sensitivity being the crucial modal condition. Not being closed under known entailment is one of the crucial features of sensitivity. Thus, checking is clearly not closed under known entailment according to this sensitivity-based checking account. This fact is acknowledged by Baumann, and so he raises the slightly different question of whether checking in the sense that he prefers, which is not modally enriched, much more internalist and perhaps not even factive, is closed under entailment. After running through classical schemes of knowledge closure and their problems, Baumann (forthcoming) proposes the following closure principle for checking:

(Full Checking Closure) If S has checked that *p* and has come to believe that *q* on the basis of competent deduction from *p*, then S has checked whether/that *q* – but not if S could only check that *p* because they presupposed and took for granted the (so far) unchecked *q*.^2^

Baumann regards this as an independently plausible principle of checking closure. I already discussed Baumann’s internalist version of checking above, so let me grant for the sake of argument his checking account and reflect on whether it meets this closure condition. According to the checking account of KC, S can check that there is a zebra in the pen if S has the corresponding intention and uses a method that is sensitive with respect to there being a zebra in the pen. One need not presuppose and take for granted that there is not a painted mule in the pen for checking successfully that there is a zebra in the pen. However, I accept that checking is not closed under known entailment; thus, S cannot check that there is not a painted mule in the pen via deduction from there being a zebra in the pen, because the method is sensitive to the second but not to the first proposition.

What verdict does Full Checking Closure, paired with Baumann’s internalist sense of checking, deliver concerning the zebra case? This depends on whether one can internalistically check whether there is a zebra in the pen without presupposing and taking for granted that there is not a painted mule in the pen. Baumann (forthcoming) suggests that one cannot when noting: “Did I really check whether that animal is a cleverly disguised mule by looking at it, using my ordinary zoo visitor’s knowledge as background and inferring that it is not a cleverly disguised mule? There seems something wrong with this kind of inference.” As I understand Baumann, zebra cases fulfill his Full Checking Closure but only in the sense that the exception clause applies; i.e., even if S has checked that the animal in the pen is a zebra and competently deduced from that that it is not a painted mule, S thereby did not check the latter because S could not have checked that the animal in the pen is a zebra without presupposing and taking for granted that it is not a painted mule.

According to the account of KC, checking is not closed under known entailment; Baumann suggests that internalist checking is closed, but with some important restrictions. Do these two accounts really deliver different verdicts? As I understand Baumann, his account does not rule out that one can internalistically check that the animal in the pen is a zebra by presupposing and taking for granted that it is not a painted mule. It only rules out that one can then check via deduction that the animal is not a painted mule. Thus, we can Baumann-check that the animal is a zebra but we cannot Baumann-check that it is not a painted mule. Full Checking Closure is, nevertheless, fulfilled, but only in virtue of the restrictions imposed by Baumann. In KC, I defend the view that checking is not closed under known entailment in a traditional unrestricted sense. Baumann, as I understand him, suggests that checking is closed in a restricted sense. Given that both accounts deliver the same verdicts in zebra cases (and presumably also in alternative cases), the difference between these two approaches does not concern closure puzzles.

As a final note on closure, Baumann (forthcoming) points out that if checking is contrastive, as he suggests, “then closure principles for checking will have to take this into account. Jonathan Schaffer has given a rather detailed account for how to formulate closure for contrastive knowledge (see Schaffer, 2007). Something of that kind would have to be done for contrastive checking.” I agree with Baumann on the possibility of pursuing this project, but it seems far more pressing if someone accepts closure for checking than if someone rejects it, as I do.

### Generality and Heterogeneity

The generality problem is one of the most notorious problems for externalist knowledge accounts. Since KC defends an externalist account for checking, we have to discuss whether checking suffers analogously from the generality problem. I argue in KC that this is not the case, because the method is specified by the intentions of the checking subject. Baumann argues that this solution still leaves enough indeterminacy for some kind of generality problem, since checking subjects are plausibly often not able to provide a complete description of the method used, which might be necessary to determine the modal profile of the method and whether it is a checking method. I agree with Baumann about these practical obstacles to determining which method the subject intended to use, and I acknowledge these problems in Chapter 3.8 of KC. However, I think the problems are different in nature. While it is *in principle* problematic to determine the method in cases subject to the generality problem, I see only *practical* limitations and problems in case of checking. If a subject has complete knowledge about her intentions, the issue can be resolved while the generality problem, which does not rely on any intentions, would still remain.

In Melchior (2015), I argue that, when it comes to higher-level knowledge that one’s own beliefs are true and not false, we face the problem that some of these beliefs are insensitive but beliefs in very similar propositions are sensitive, calling this the “heterogeneity problem.” This problem seems to generalize to other cases, illuminating a certain instability of sensitivity.^3^ In a final note, Baumann presents a further version of the heterogeneity problem. He argues that in Kripke’s (2011) barn case, S’s belief that the barn is red is sensitive according to the standard reading and therefore can constitute knowledge according to a sensitivity account. However, when S comes to believe that there is a red barn via conjunction introduction from “There is a barn” and “That thing is red,” then S plausibly does not know because she drew the inference from a proposition that she does not know according to sensitivity accounts, namely, that there is barn.^4^

This is a smart observation and I agree with Baumann on this version of the heterogeneity problem for *knowledge*. The crucial question is whether it also poses a problem for KC’s sensitivity account of checking. In KC, I distinguish between deductive checking in a narrow sense, which is specified as the method of consulting the very same sources, and deductive checking in a wide sense, which involves consulting the very same sources that deliver the premises. Deduction in a narrow sense is insensitive, but deduction in a wide sense can be sensitive. Thus, I reject deduction in a narrow sense as a method for checking but accept deduction in a wide sense. In cases of deduction in wide sense, S uses the same source and then draws deductive inferences. In the nearest possible worlds where S uses eyesight plus deduction to determine whether there is a red barn in front of her and there is no red barn in front of her, S’s method does not indicate that there is a red barn in front of her. Thus, S’s method for determining whether there is a red barn in front of her is sensitive regardless of whether it involves deduction via conjunction introduction or not. Thus, the particular heterogeneity problem that Baumann insightfully introduces for knowing does not arise for the checking account of KC.

## Kelly Becker: Sensitivity: Checking into Knowing?

I am very thankful to Kelly Becker for his excellent comments on KC. He knows as much as anybody about modal epistemology, and his outstanding monograph *Epistemology Modalized* (Becker, 2007) was one of the central key readings for my own work. Becker (forthcoming) provides a battery of suggestions concerning the sensitivity account of checking developed in KC. In this reply, I will first focus on his criticism of KC’s solution to the generality problem, second on his suggestion to extend the explanatory power of the proposed checking account, third on his interpretation of the sensitivity principle, and fourth on his reflections on the sensitivity of Moorean reasoning.

### The Generality Problem

The generality problem for knowledge is the problem of specifying the method used during a belief forming process. In KC, I argue that my checking account does not suffer from the generality problem because the method is specified by the intentions of the checking subject, i.e., by the method that the subject intentionally chooses to use. A particular method token belongs to (infinitely) many method types, but the checking subject intends to use a particular method under a particular cognitive representation, and so the generality problem is avoided. This also holds for contrastive checking, where the subject has a specific intention to check whether* p* instead of a particular alternative* q* is true. Becker (forthcoming) presents the following example in order to argue that this leads to implausible consequences:

Suppose Bugs wants to check whether it is Tom or Jerry hiding behind the tree. Bugs forms the following intention: Go look behind the tree and see whether there’s a gray cat who looks like Tom or a brown mouse who looks like Jerry. When he sees a gray cat, he’s checked that it’s Tom and not Jerry. If it were Jerry and not Tom, his method wouldn’t indicate that it’s Tom.

That seems about right. But doesn’t Bugs’s method also successfully check that it isn’t Daffy hiding behind the tree? Bugs’s intention is to check whether it is Tom or Jerry. Perhaps he assumes it’s one or the other, but for all he knows it could be someone else, or nobody at all. But after the fact, has he not checked that it’s not Daffy? Elmer asks, “Is Daffy behind the tree?” Bugs can say, “No, I checked.” But that wasn’t part of his intention, so it wasn’t part of his method. I supposed one could say that when Elmer asks, Bugs can then form the intention to determine whether it was Tom, Jerry, or Daffy and consult his experience-based memory of its being Tom and thereby have checked that it isn’t Daffy. That is, one could say generally that when new questions arise, old checking methods can be revised with the new intentions. But I don’t think that’s what Bugs does. He only ever intended to determine whether it’s Tom or Jerry, and implementing a method to find out, he also checked that it wasn’t Daffy. He’s also potentially checked—and counts as having checked if the relevant questions arise—many other things, such as that there’s not a Tasmanian Devil or a basketball hoop or a broken scooter behind the tree.

In this passage, Becker argues for the following claim: If S had the intention to check whether* p* or *q* is true, and comes to know that *r* is true instead of *p* or *q*, then, when asked, S can correctly claim to have checked that *r* is true. However, according to the checking account of KC, S did not check that *r* is true in this case, because having the intention to check that *r* is true (or an alternative *t*) is necessary for successfully checking that *r*.

I understand the intuition that Becker stresses, but I am not sure whether I share it. First, there is some oddity in claiming that S checked that *r* without ever having the intention to check that. Take the distinction between investigating and discovering in science. If a scientist S investigates a certain phenomenon* p* but discovers another phenomenon *q*, then it seems false to say that S investigated whether* q* is true despite her discovery of *q*. Rather, we say that S investigated* p* and luckily discovered *q*. By stressing this analogy, one could straightforwardly reject Becker’s suggestion that S checked that *r* by investigating* p* or *q* and thereby discovering *r*. Second, even if one shares, to a certain extent, Becker’s intuition that S can check that* r* by having the intention to check whether* p* or* q* is true, one might be able to explain this intuition away for the sake of saving a unified account of checking. Becker discusses the possibility of describing the method of checking whether* r* is true in his example as consulting memory. He rejects this option, arguing that it does not correctly describe the processes. However, there must be some kind of memory and inference involved in S’s report, given that S is later confronted with the possibility that *r*. Thus, I do not see why this possibility should be so easily dismissed. Furthermore, one can also reply to Becker that we sometimes use loose talk about checking from an ex post point of view, focusing on the outcome of the checking process and tending to ignore the intentions of the checking subject. Thus, in a loose sense (but only in a loose sense), we can say that S checked that *r* is true by having the intention to check whether *p* or *q* is true. Moreover, there are also methodological reasons for rejecting Becker’s suggestion that S can check that* r* when having the intention to check whether* p* or *q* is true. From an ex ante point of view, before the checking procedure delivers a particular outcome, it seems highly implausible to claim that S checks whether* r* is true by having the intention to check whether* p* or *q* is true. But in order to acquire a unified picture of ex ante and ex post reports about checking, we should accept the same mechanisms for ex post reports. Accordingly, we cannot say from an ex post point of view that S checked that* r* is true when having had the intention to check whether* p* or *q* is true. I am not sure whether KC’s solution to the generality problem really has some counterintuitive implications, but I think that abandoning this solution because of Becker’s objections is a price too high to pay.^5^

### Low-Stake Contexts

In the second part of KC, I use the sensitivity account of checking I developed to explain knowledge puzzles by relying on intuitions about knowing and checking. The following principle KSAC captures the central connection between our intuitions about knowing and our intuitions about checking:

KSAC

In contexts of checking, when we raise the question whether* p* (or an alternative *q*) is true and deliberate about methods for settling this question, we tend to think that we do not know that* p* via strongly insensitive methods, especially not via monotonous methods. In other contexts, this tendency does not apply. (KC, 142)

In KC (143), contexts of checking are characterized more specifically as the conjunction of the following three tendencies:

1. In contexts where S_1_ raises the question whether* p* is true and deliberates about methods for settling this question, S_1_ thinks she does not know that* p* via strongly insensitive methods.
2. In contexts where S_1_ raises the question whether* p* is true and deliberates about methods for settling this question, S_1_ thinks that S_2_ does not know that* p* via strongly insensitive methods.
3. In contexts where S_1_ thinks that S_2_ raises the question whether* p* is true and that S_2_ deliberates about methods for settling this question, S_1_ thinks that S_2_ does not know that* p* via strongly insensitive methods.

Becker (forthcoming) challenges this characterization of checking contexts by arguing that sometimes I characterize checking contexts too loosely for my own purposes and sometimes too tightly “where loosening them up a little creates opportunities for even greater explanatory power.” Note at this point that KSAC can be interpreted in different ways. KSAC states that checking contexts are necessary and sufficient for the tendency to think that we do not know that* p* via strongly insensitive methods. According to a strong reading, KSAC also provides necessary and sufficient conditions for entering a checking context, namely, by raising the question whether* p* (or an alternative *q*) is true and by deliberating about methods for settling this question. According to a weak reading, these are only sufficient conditions. In KC, I do not address this ambivalence of KSAC, but in light of the objections raised by Becker (forthcoming) and David (forthcoming), I prefer here the weaker reading. Accordingly, also the tripartite version of KSAC should be understood as only expressing *sufficient* conditions for entering a checking context. This suffices for explaining closure puzzles and the skeptical puzzle in terms of intuitions about sensitive checking. This weak reading of KSAC does not rule out that there might be other mechanisms for entering a checking context. Hence, any form of loosening checking contexts, as suggested by Becker, is in line with the weaker reading of KSAC.

Let me first address Becker’s case that the provided definition of checking contexts is too wide. Becker presents a low-stake case, where the practical interests of Keith and anybody else whose interests might be relevant are low. Becker (forthcoming) supposes that, in this case, the protagonist Keith “raises the question, deliberates about how to settle it, then asks a friend who says, ‘Well, most banks are open on Saturday,’ and concludes that the bank will be open.” This is a checking context, since condition (3) for KSAC is fulfilled for the reader. Since the method of consulting a friend’s testimony plus induction is insensitive, KSAC predicts that we have the intuition that Keith does not know. However, this verdict conflicts with the intuition that subjects know in low-stake cases. Thus, we face a tension of conflicting intuitions.

Becker suggests to resolve this tension by defining checking contexts more strictly. In KC, I define checking contexts more loosely than checking itself by arguing that raising a question and deliberating about a method are already sufficient for entering a checking context without the actual intention to check. For this reason, Keith is in a checking context in Becker’s case despite not actually checking. But if we define checking contexts more narrowly, such that intentions to check, and perhaps also checking actions, are necessary, then Keith is not in a checking context and we should not have the intuition that he does not know. This is in line with our low-stake intuitions and the tension is resolved, as Becker correctly notes.

How can we evaluate Becker’s proposal? First of all, it is true that the predictions of KSAC and low-stake/high-stake intuitions can come apart. KSAC is more closely related to a relevant-alternative contextualism, where raising the question whether* p* (or *q*) is true makes the alternative ~ *p* (or ~ *q*) salient, and they then must be ruled out via a method that is sensitive with respect to that alternative. I chose raising the question and deliberating about methods for settling it as sufficient conditions for checking contexts in order to keep as close as possible to knowledge puzzles based on alternatives, such as lottery alternatives or deception alternatives, which are typically made salient by deliberating about it. What is the correct verdict in Becker’s case? I am not sure how to answer the question, but I still feel the intuition that Keith does not know in this low-stake case that the bank is open because of raising the question and deliberating about methods for settling it. This, however, commits me to rejecting some straightforward intuitions about knowledge in low-stake cases, which is an intriguing point that Becker makes.

Secondly, let me discuss Becker’s suggestion that the proposed definition of checking contexts is too narrow. In KC (157f), I discuss the following case:

Soy Oil

S has statistical knowledge that 1% of pizzas contain soy oil. S is served a pizza in a restaurant that does not contain soy oil. We think that S knows that the pizza does not contain soy oil based on S’s statistical evidence if not much hinges on it. However, we also think on the same basis that S does not know, if S is allergic to soy oil. Moreover, we think that S would know that the pizza does not contain soy oil (only) if S has additional evidence e.g. if S had asked the chef.

S raises a question about soy oil neither in a low-stake context nor in a high stake context, so KSAC cannot explain the context shift in that case. In KC, I argue that KSAC can explain shifting intuitions in lottery alternatives and deception cases, but I also admit that it cannot explain all low-stake/high-stake cases. Becker argues that, in this case, we still have the intuition that S *should* have checked and that, in this respect, the explanatory power of KSAC should be extended.

This looks like a promising suggestion since the method of asking the chef in the example is plausibly sensitive. A generalized account about checking contexts based on this approach could hold that S enters a checking context if S or someone else raises a question and deliberates about methods for settling it *or* if S is in a situation that she should check with respect to some specific goal. But with respect to what goals should S check? An extended account of checking contexts has to answer this question. One plausible answer for Soy Oil is that S should check with respect to the goal of staying healthy. However, this goal can surely not be generalized to all cases where we intuit that S should have checked. One might argue that, as part of a theory that explains knowledge or knowledge intuitions, the goal should be knowledge-related. So, S should have checked with respect to the goal of acquiring knowledge. This seems in line with the intuition that S does not know in checking contexts. I find Becker’s suggestion of modifying checking contexts inspiring. Moreover, it is in line with the weak reading of KSAC, which only provides sufficient conditions for entering a checking context and which is, therefore, open to further mechanisms. Nevertheless, whatever an extension of the definition of checking contexts might look like, it has to be noted that I also discuss in KC cases that are not covered by KSAC and that would also not be covered by this extension, for example, when intuitively meta-knowledge is required for knowledge in high-stake cases.

### Sensitivity

In KC, I argue that sensitivity marks a crucial distinction between knowing and checking. While checking essentially requires sensitivity, knowing plausibly does not (at least in some contexts). Becker (forthcoming) turns to the sensitivity condition and sketches a project that he summarizes as follows:

My aim in this section [is] not, perhaps despite appearances, to defend the sensitivity condition on knowing. My aims were more limited, namely, to show that the sensitivity conditional itself, unanalyzed, unrelativized, and unadorned, speaks to a compelling idea about knowledge—that, in many, many cases, we don’t know that* p* if we would believe that* p* even if it were false. This tells us something about the nature of knowledge—of a lot of knowledge, though not all—that the safety principle does not.

Becker analyzes classical cases discussed in the literature about sensitivity along these lines, such as Nozick’s (1981) grandmother case, Sosa’s (1999) trash chute case, and Vogel’s (2007) heartbreaker case.^6^ He suggests that our intuitions about the relevant counterfactuals are strong and stable and that the counterfactual simpliciter has more explanatory power than when it is accompanied by further supplements about the method used and that “objections and replies are all quibbles.”

Becker’s reflections are intriguing. He focuses on sensitivity as a necessary condition on knowledge correctly stating that “safety does not cast much light on the nature of knowledge.” In KC, I develop a sensitivity-based account of checking. Although I argue that safety is also necessary for checking, sensitivity and not safety are the crucial necessary conditions doing the explanatory work about the nature of checking. Thus, safety does not cast much light on the nature of *checking* either, according to KC. Nevertheless, Becker’s suggestion to consider counterfactuals simpliciter without referring to the method used is not applicable to checking. Checking is genuinely an action, in contrast to knowing, which is a state, and the action involves using a particular method. Thus, using a method is directly incorporated into the concept of checking, in contrast to knowing. Therefore, considering counterfactuals without the method is a viable option for modal accounts of knowledge, but not for checking.

In KC, I also discuss Kripke’s (2011) famous barn case against knowledge. Kripke argues that, according to Nozick’s sensitivity account of knowledge, S can know that there is a red barn in front of her without knowing that there is barn in front of her, which Kripke and many others regard as an absurd consequence. This also poses a problem for KC’s sensitivity account of checking, since it also seems problematic to claim that S checked that there is red barn without checking that there is barn. I argue in KC that, from an ex ante point of view, it is plausible to claim that S is in a position to check whether there are red barns in the field but not in a position to check that there are barns.^7^ In KC, I discuss various solution to Kripke’s barn case. First, from an ex ante point of view, it seems plausible to claim that Barney is in a position to check that there are red barns but not that there are barns. Second, checking that there is a barn by checking that there is a red barn and checking that there is barn based on background knowledge about the existence of non-red barn façades are also sensitive. Nevertheless, I am committed to the accepting the claim that S checked that there is a red barn but did not check that there is a barn. In this very special case, I bite the bullet for saving a unified sensitivity account for checking.

### Moorean Reasoning

We have conflicting intuitions about bootstrapping, the process of coming to believe that a source is reliable via information from this very source.^8^ On the one hand, bootstrapping is intuitively a flawed epistemic process. On the other hand, basic knowledge (i.e., knowledge via a source without knowledge that the source is reliable) and knowledge via induction are plausible, and jointly they allow for knowledge via bootstrapping. In KC, I provide an explanation for these conflicting intuitions. I argue that bootstrapping is an insensitive method and, therefore, not a method for checking whether the skeptical hypotheses are true. This insensitivity explains our intuitions that bootstrapping is epistemically defective. However, I also suggest that there are, despite these intuitions, good reasons to accept that we can know via a complex form of bootstrapping that the skeptical hypotheses are false.

Becker points out that an externalist disjunctivist interpretation of methods allows for a sensitive interpretation of Moorean reasoning. He develops further a suggestion made by Black (2002) that methods should not be individuated from the inside, as Nozick suggests, but externally, from the outside. Suppose S’s belief forming method is seeing. According to this conception, we cannot correctly say that, in the nearest possible worlds where* p* is false and S uses the same method as in the actual world, seeing that *p*, S comes to believe that *p*, because seeing is factive. Thus, S’s belief formed via seeing is sensitive. Becker does not aim at showing that an externalist interpretation of sensitivity is correct. His dialectical position is more subtle. He summarizes his dialectical position concerning disjunctivist Moorean reasoning, [D]Moore, as follows:

I raise this issue not because I accept [D]Moore’s views. I don’t. I think my belief that I’m not a BIV is straightforwardly insensitive. If I were a BIV, I would think I’m not. That’s it. I cannot discriminate the actual world from BIV worlds. One of the main points of the previous section is that we should be wary of appealing to (various ways of construing and individuating) methods and backtrackers to rescue or criticize sensitivity. But if one wants to play that game, what one might find interesting about the upshot of this final section is that, whereas, typically, self-styled neo-Mooreans reject sensitivity in favor of safety, perhaps there’s no reason to. (Becker, forthcoming)

I completely agree with Becker that the discussion about safety and sensitivity is one-sided. For example, it is often said that one central advantage of safety over sensitivity is that it accepts knowledge closure, although safety violates knowledge closure in the same highly implausible Kripke cases which have been used against sensitivity.^9^ Moreover, I agree that there is a tension between a modal knowledge account that is externalist with respect to the modal conditions and an internal specification of the methods used. Thus, sensitivity accounts of *knowledge* can argue for an externalist interpretation of methods (although Black’s externalist sensitivity account faces problems that I address in Melchior (2015) and in KC). Becker’s point about knowledge is well taken.

But can this approach of sensitive bootstrapping via externally specified methods also be applied to the checking account of KC? Such an application had a serious impact on the argumentation of KC, since I argue there that the insensitivity of bootstrapping explains the tendency to judge that we cannot know via bootstrapping. However, if bootstrapping is sensitive, then this explanation is not available. We could fix the problem by claiming that the false judgment that bootstrapping is insensitive explains the tendency to judge that we cannot know via bootstrapping, but this weakens the explanatory power by adding a further component that has to be defended.^10^

However, an externalist specification of methods, as Becker discusses, does not fit the checking account of KC. In KC, I argue that checking is an intentional action and that the method is determined by the intentions of the checking subject. According to disjunctivism, methods are (at least partly) characterized from the outside. Intentions, in contrast, are always characterized internalistically. Thus, the ordinary person and the BIV apply the same methods according to the specification of methods provided in KC, in contrast to an externalist specification of methods.

I share Becker’s concerns about the objections to sensitivity in favor of safety in the context of knowledge accounts. Moreover, I agree that, with knowledge accounts, it might be plausible to use counterfactuals directly without considering the method. However, I doubt that this intriguing project can also be applied to the sensitivity-based checking account of KC.

## Marian David: Analytic Epistemology and Armchair Psychology

I have known Marian David for many years as a deep thinker, a superb commentator, and an extremely well-read philosopher. He has given extensive comments on the whole manuscript of KC, commenting on some chapters twice. David focuses on part 2 of KC, which is devoted to the relation between knowing and checking, and addresses three main issues, as he calls it, which I will address in turn.

### First Issue

In part two of KC, I use the sensitivity-based theory of checking developed in part one for explaining enduring knowledge puzzles, in particular puzzles about knowledge closure. I argue that, in certain contexts of checking which are specified in detail in KC, we tend to think that knowledge that* p* requires a checking method, i.e., a method that is sensitive concerning *p*, whereas in other contexts, we do not tend to think that such a method is required for knowing. Thus, this second part of KC deals to a large extent with our *intuitions* about knowledge, i.e., about what we *think* about knowledge in different contexts. In this respect, it essentially contains psychological hypotheses. David (forthcoming) addresses the type of psychological assumptions made in KC and raises the following questions:

1. What is the psychological mechanism? Is there some kind of migration plus infection involved here […]? Is it that, in contexts of checking, some of our intuitions about what’s necessary for checking migrate over to our judgments about instances of knowledge and affect-infect them?
2. Is there some sort of psychological associationism in the background here?
3. Can one test such hypotheses empirically? What would that involve?
4. Does Melchior agree that, at least in principle, one should test such hypotheses and that, as long as this remains undone, the case made in the second part of the book remains incomplete?
5. What about competing hypotheses?

These are insightful questions about the nature of the central hypotheses in part 2 of KC. Let me address them step by step.

- (1) and (2): The central principle KSAC that I defend in KC has it that, in checking contexts, we tend to think that a sensitive method is required for knowing, whereas in other contexts, this tendency does not apply. I apply this principle without providing a deeper analysis of the underlying mechanisms. My impression is that the underlying mechanism is a transfer of intuitions about epistemic *defects* from checking to knowing. We think, correctly, that strongly insensitive methods are epistemically defective in the sense that they are flawed methods for checking and then conclude that they are also flawed methods for knowing. For example, we think, in checking contexts, that monotonous methods are epistemically defective with respect to the goal of checking and then reason by analogy, correctly or not, that these methods are also flawed concerning the goal of achieving knowledge.
- (3): Yes, I think that these hypotheses can be empirically tested by applying the methods of experimental philosophy.^11^ But let me make some general remarks about philosophical analyzes of intuitions. When dealing with intuitions in philosophy, we always face the challenge of carefully steering between the Scylla of providing a purely descriptive theory of intuitions without any systematic and philosophical value and the Charybdis of developing a purely prescriptive theory which does not take our common sense intuitions into account. This is a challenge which not only KC but also all alternative philosophical theories face. To a certain extent, no philosophically interesting systematic theory can do justice to the whole body of incoherent intuitions.
- (4): I also accept David’s fourth suggestion. The second part of KC is speculative, as are other theories aiming to explain conflicting knowledge intuitions, including contextualist theories or subject-sensitive theories.^12^ In principle, these theories are subject to empirical verification or falsification, and consequently, they should be tested. Without testing, explanations of intuitions remain to a certain extent incomplete, as David correctly points out.
- (5): It is, indeed, helpful to formulate competing hypotheses, and the psychologically speculative claims of KC should not only be tested in isolation but also with respect to alternative theories. The theory about knowledge intuitions discussed in KC, KSAC, has it that, in checking contexts, we tend to think that a sensitive method is required, whereas in other contexts, this tendency does not apply. In order to be persuasive, KSAC must explain our intuitions more adequately than other theories, which will also depend on which intuitions we actually have. I argue in KC that KSAC can explain closure puzzles based on deception propositions and lottery propositions particularly well, because the cases are usually set up in a way that the beliefs in the ordinary propositions are sensitive but the beliefs in deception propositions and the beliefs in lottery propositions are insensitive. However, I also admit in KC that KSAC cannot explain all knowledge puzzles; in particular, some puzzles concerning low-stake cases and high-stake cases cannot be adequately addressed by KSAC. Alternative cases and theories about low-stake cases (LOW) and high-stake cases (HIGH) mentioned in KC (158) are as follows:
  - (1) We think that S’s evidence e is sensitive, and we think in LOW that S knows that *p*, but we think in HIGH that S has to acquire additional evidence for knowing.
  - (2) S’s evidence e is not sensitive. We think in LOW that S knows that *p*, and we think in HIGH that S would know via evidence that is also not sensitive.
  - (3) S has externalistic but not internalistic evidence e, and we think in LOW that S knows that *p*, but we think in HIGH that S must have internalistic evidence or meta-evidence for knowing that *p*.

In his comments, David (forthcoming) suggests the following competing hypothesis:

Formulating a competing hypothesis would help with testing. Here is a (rather vague) suggestion. Raising the question whether* p* is true puts us into a defensive debating mode; it makes us consider what sort of considerations would convince an opponent who challenges a statement we made (“You say it’s a zebra. How do you know it’s not a cleverly painted mule?”). I am not entirely sure whether this would amount to a hypothesis seriously competing with the one Melchior has suggested, but it might be a start.

David is correct concerning the comparison of KSAC with competing hypothesis, but I am not sure whether his suggested alternative is particularly promising. If persuading an opponent that the animal in the pen is a zebra and not a painted mule tends to require methods of discriminating zebras from painted mules, which I regard as a plausible criterion, then a sensitive method concerning the animal being a zebra and not a painted mule would be required.^13^ Thus, David’s competing hypothesis collapses into the view defended in KC, or at least comes close to it. However, alternative interpretations of David’s suggestion are also possible.

### Second Issue

The second point that David makes is that some psychological explanations of KC are as he calls it “farfetched” and, therefore, implausible. He discusses the following case of KC (165):

Suppose we think that our evidence is sensitive for o but strongly insensitive for ~d. According to KSAC, we think that we know that o no matter whether we are in a checking context for o or not. Moreover, we think that we know that ~d when we are not in a checking context, i.e. when we are not raising the question whether ~d is true.^14^ However, when we raise the question and deliberate about methods for settling it, we no longer think that we know that ~d based on the evidence we have.

David (forthcoming) objects that this interpretation cannot be correct if taken literally:

Can this be taken seriously? Does Melchior really mean to say that, looking at the zebra case, someone actually thinks: “S knows that the animal in the pen is not a mule cleverly painted to look like a zebra”? I don’t think so. Melchior must be using “think” in a rather loose sense here.

On this point, I disagree with David’s interpretation of the case presented in KC. In KC (165f), I analyze the zebra case as follows:

ZEBRA: S believes via eyesight and background knowledge about zoos in general that in the pen is a zebra. This evidence is sensitive with respect to ‘In the pen is a zebra’ because in the nearest possible worlds where there is no zebra in the pen, S’s eyesight plus background knowledge does not indicate that in the pen is a zebra. However, S’s eyesight plus background knowledge is not sensitive with respect to ‘There is not a painted mule in the pen.’ We believe these facts about the (in)sensitivity of S’s evidence. Accordingly, we believe that S knows that in the pen is a zebra no matter whether we raise the question whether this is true and deliberate about methods for settling this question or not. Moreover, if we do not raise the question whether in the pen is not a painted mule, we believe that S also knows this. But if we raise this question and deliberate about methods for settling it, then we think that S does not know via eyesight and background knowledge about zoos in general that in the pen is not a painted mule.

David seems to suggest that I am in some sense committed to accepting that S knows via eyesight and background knowledge that the animal in the pen is not a painted mule. According to KC’s interpretation of the zebra case, we judge that S knows that the animal in the pen is a zebra due to the sensitivity of S’s observation of a zebra, regardless of whether we raise the question whether the animal in the pen is a zebra or not. This part does not commit me to accepting that S knows that the animal in the pen is not a cleverly disguised mule. Moreover, when we raise the question whether the animal in the pen is a disguised mule, then we judge that S does not know that it is not a painted mule. (The same holds when we raise the discrimination question whether the animal is a zebra *or* a painted mule, which I discuss as an alternative explanation of deception cases in KC). Thus, I do not see why I am committed to accepting that there are contexts where we judge that S knows that the animal in the pen is a zebra and not a painted mule *on the basis of observation and background knowledge*. This would be the case only if we could judge that S knows that in the pen is a zebra and not a painted mule without thereby (implicitly) raising the question whether the animal is a painted mule (or a zebra). However, I deny this, at least for contexts of philosophical reflections. Thus, while David’s objection can be met, it illuminates the important point that contexts for checking can rather easily occur, in particular in philosophical analyses.

Based on his criticism, David (forthcoming) proposes the following solution to the zebra problem:

It would be more defensible to describe what is going on in terms of what we think combined with what we are thereby committed to, but don’t think. Concerning the zoo scenario, we think that S knows that the animal in the pen is a zebra. Given that we tend to be committed to knowledge closure, thinking that S knows that the animal in the pen is a zebra commits us to judging that S knows that the animal in the pen is not a mule cleverly painted to look like a zebra. But we don’t think that, and don’t judge that: in fact, we judge the opposite. Hence the puzzle. Of course, this more defensible talk in terms of commitments evades the psychological issue. It does not help at all with identifying psychological mechanisms. It seems to me that Melchior’s loose use of the term “think” at these occasions is a symptom of his reticence with respect to invoking any psychological mechanisms.

As David correctly notes, this solution does not explain any psychological thesis about knowledge intuitions, which the account in KC addresses. Since I think that this account in KC does not suffer from the problems that David raises, I prefer the explanation of KC over his alternative.

### Third Issue

The last point that David (forthcoming) raises concerns the question of who, if anybody, is in a context of checking in the cases analyzed in KC, which David correctly characterizes as imaginary scenarios. “Now, concerning the Zebra case, it seems to me that there isn’t actually anyone who is in a context of checking, as such contexts are described by Melchior.” David’s second and third issues both address the question of how to specify checking contexts, pressing me to provide a wider definition of checking contexts. David (forthcoming) points out that “[t]he person, S, within the imaginary scenario is not in a context of checking.” David continues:

It is a puzzle for us, the readers. Why are we, the readers, inclined to judge the way we do?

Given how Melchior conceives of checking contexts, we, the readers, are not in a context of checking either. We don’t raise the question whether the animal in the pen is a zebra, or deliberate about methods for settling this question. That the animal in the pen is a zebra has been stipulated to be true (or stipulated to be pretend-true) by the setting of the imaginary case. So, with respect to the zebra case, I don’t find anyone who is in a checking context as such contexts are conceived by Melchior. […]

Melchior specifies the conditions that must obtain for someone to be in a checking context. A person is in a checking context, if she raises the question whether* p* is true, deliberates about methods for settling this question, and makes a knowledge judgment about herself, or about some other person; or the person believes about some other person that that person raises the question whether* p* is true, deliberates about methods for settling that question, and makes a knowledge judgment about herself. As far as I can see, none of these conditions obtain with respect to the readers of Zebra cases whose judgments about what S knows and does not know inside the imaginary case are supposed to be explained.

Let me recapitulate how checking contexts are specified in KC. In KC (143), I define checking contexts as the disjunction of the following three cases:

1. In contexts where S_1_ raises the question whether* p* is true and deliberates about methods for settling this question, S_1_ thinks she does not know that* p* via strongly insensitive methods.
2. In contexts where S_1_ raises the question whether* p* is true and deliberates about methods for settling this question, S_1_ thinks that S_2_ does not know that* p* via strongly insensitive methods.
3. In contexts where S_1_ thinks that S_2_ raises the question whether* p* is true and that S_2_ deliberates about methods for settling this question, S_1_ thinks that S_2_ does not know that* p* via strongly insensitive methods.

Any of these three cases specifies a checking context. It is true in zebra cases that the protagonist is not supposed to raise the question whether the animal in the pen is a zebra and to deliberate about methods for settling it. Therefore, zebra cases are not instances of (1). Moreover, we, the readers, are not supposed to think that the protagonist does so. Therefore, zebra cases are also not instances of (3). Thus, zebra cases, if contexts of checking, have to be instances of (2). For acquiring this result, some modification is required. Given that we assume that S *knows* that the animal in the pen is a zebra, it is correct to say that we presuppose that the animal *is* a zebra.^15^ But given that we presuppose that the animal in the pen is a zebra, we cannot correctly say that we raise the question whether the animal in the pen is a zebra. Thus, the first part of David’s disjunctive objection, claiming that we “don’t raise the question whether the animal in the pen is a zebra, or deliberate about methods for settling this question,” is correct. However, it seems to me clearly the case that in the context of philosophical reflections, we deliberate about methods for settling the question whether the animal in the pen is a zebra or a painted mule or for discriminating the one from the other.^16^ Thus, the second part of David’s disjunctivism, that we do not deliberate about methods for settling the question, is false.

David is correct that the original formulation of checking contexts does not deliver the desired results. His reflection on the zebra case and related philosophical cases illuminates that, in some cases, the deliberation about methods for settling a question is sufficient for entering a checking context. Raising the question itself is not required. In my replies to Becker (forthcoming) above, I distinguish a strong reading of KSAC according to which KSAC provides necessary and sufficient conditions for entering a checking context, from a weak reading, according to which it only provides sufficient conditions. Given the objection raised by Becker and David, the week reading is preferable. Thus, there might also be other mechanisms than the ones listed in KSAC for entering a checking context. In this sense, KC is open to further “fine-tuning” as David suggests.

## Nenad Miščević: Curiosity, Checking and Knowing: a Virtue-Theoretical Perspective

I am thankful to Nenad Miščević not only for his comments on KC. Nenad was among the first and most influential teachers for me, opening the philosophical door to the world, and therefore, I am indebted to him for much more. Miščević focuses in his comments on the relation between checking and inquiring, connecting KC with the central ideas of his own recent work on curiosity.^17^

In the first part of KC, I develop a sensitivity-based account of checking, and in the second part, I use this account for explaining the connections between intuitions about knowing and about checking. However, I do not explore motivations for checking, leaving out the question of when a subject rationally or reasonably checks. This is subject to further work and a lacuna of KC, in particular given the recent zetetic turn in epistemology and the work on the relation between inquiry and knowledge.^18^ This is also noted by Miščević (forthcoming), when he proposes the following connection between checking and curiosity:

Guido tells us little, almost nothing, about the general context of checking. It is natural to assume that subject S would rationally turn to checking whether *p* typically, when she is curious about *p*, and she is prone to investigate a presumption in favor of *p* being true. Motivation for checking (double checking, triple checking) is thus part of the normal inquisitiveness, motivation to find out. What is the right context for checking?

Miščević provides an insightful reflection about the connection between checking and curiosity, which he regards as the central epistemic virtue. Miščević summarizes his virtue theoretic; take from Miščević (2020) on curiosity as follows:

[C]uriosity is the central motivating epistemic virtue. A human being devoid of curiosity would have little motivation to arrive at true belief and knowledge. In normal cases it is curiosity that motivates us to gain true belief and knowledge. On the usual view of motivating virtues, this would seem to make it a virtue; since it is the main spring of motivation, we should take it as the motivating epistemic virtue. After all, wanting to know whether *p* gives cognizers particular instances of *p* (or of its negation) as particular goals and the truth as the general epistemic goal. Thus, we have a truth-focused motivating virtue: inquisitiveness or curiosity having the reliable arrival at truth as the general goal. This is, I claim in my book, the core motivating epistemic virtue. (Miščević, forthcoming)

Miščević embeds his virtue theoretic account in a broader Aristotelian picture, “with virtue in the middle and vices on both sides.” He characterizes the two epistemic vices related to the epistemic virtue of curiosity as follows:

We can contrast two extremes. The first negative extreme is epistemic rashness in inquiry, reasoning, and argumentation, which goes with gullibility, uncritical acceptance and the like. The opposite vicious extreme is active inconfidence and misplaced mistrust. We can wonder about its motivation and causes. […] Here we encounter the vicious need to check. (Miščević, forthcoming)

In his paper, Miščević reflects on the question of whether and how too much curiosity, and accordingly too much checking, can preclude us from knowing. Let me address this question by starting with a rather general remark. Checking can potentially preclude us from knowing in two ways. First, if, in a checking context, the standards for knowledge rise to a level where a sensitive method is required, but such a method is not available. Second, if the checking subject suspends judgment about* p* in the context of checking and thus does not believe that *p*. In the first case, the evidence, because insensitive, is not good enough for knowing. In the second case, the evidence might be good enough for knowing, but the subject does not believe the target proposition. These two points must be carefully separated. In KC, I only reflect on the first option. I defend the principle KSAC, which has it that, in checking contexts, we tend to think that sensitivity is necessary for knowing, officially remaining neutral about whether this intuition is accurate. Nevertheless, I express some sympathies in KC with moderate invariantism as the appropriate view about the connection between knowing and checking, namely, that we can know in checking contexts via insensitive methods even though we cannot check. According to this moderate invariantism, it is actually not possible to lose knowledge in the first sense in contexts of checking, although we tend to mistakenly think in checking contexts that sensitivity is necessary for knowing.

Let me now come to the second option, neglected in KC, that checking can preclude us from knowing because we suspend judgment about the target proposition even though we might have sufficient evidence for knowing. In KC, I provide the following definition of checking:

- S checked that* p* was true via method M iff
- (1) S intentionally used M for determining whether* p* is true.
- (2) M has certain modal features with respect to* p* (especially sensitivity).
- (3) M accurately indicated that *p*.

It is argued in KC that intentionally using a method for determining whether* p* is true requires entertaining a proposition, but I do not say anything about whether this attitude requires suspension of judgment about *p*. In fact, there is a lively discussion in recent epistemology about the relation between inquiry and suspension of judgment. Some argue that suspension of judgment (or a related interrogative attitude) is necessary for inquiry (Friedmann, 2019; Kelp, 2021) while others oppose this view (Falbo, forthcoming). Furthermore, it is also argued that suspension of judgment is not only necessary but also sufficient for inquiry (Friedmann, 2017). What is the connection between suspending judgment, checking, and knowing? If suspending judgment is necessary for checking and suspending judgment about* p* rules out believing that *p*, then S cannot believe that* p* when checking whether *p* is true and, consequently, S cannot know that *p* when checking that *p*. In this case, checking that *p* rules out knowing that *p*. I have not argued in detail for the claim yet, but I find it most plausible that S can check whether *p* is true despite believing that *p*; i.e., suspending judgment is not necessary for checking (and I also think it is not sufficient). Consequently, I think that checking does not preclude one from knowing; i.e., S can check that *p* despite believing that *p* and despite having evidence that is sufficient for knowing that *p*. Nevertheless, in many cases of checking, S will plausibly suspend judgment about the target proposition.

Whether too much curiosity and too much checking precludes us from knowing is the question in which Miščević is interested. Too much curiosity can preclude us from knowing if the kind of curiosity involved requires suspension of judgment. Checking, according to the account of KC, does not require initial suspension of judgment—it only requires raising a question, and I assume that one can raise a question without suspending judgment. Thus, checking does not always preclude one from knowing. One can check that *p* despite believing that *p* and despite possessing sufficient evidence for knowing. Too much doubting precludes one from knowing, as Miščević correctly points out. He also assumes that too much curiosity precludes one from knowing, and I agree with him if the kind of curiosity involves suspension of judgment. However, too much checking itself does not preclude one from knowing. It only does so if it involves suspension of judgment, which is not always involved in checking.

Miščević (forthcoming) endorses the view that any curiosity concerning deception possibilities is already reflective, and he suggests that this also holds for checking:

What is in the book described as a motivation to check seems to me a particular kind of curiosity. To stay with examples from the book, consider ordinary curiosity about the animal at the exhibit. The first order curiosity asks what animal it is or whether it is a zebra. Checking happens at a higher level: if Thomas doubts and asks himself whether it is really a zebra or whether he has misperceived the animal, he climbs to a higher level. The book specifies that he intentionally uses the chosen method to find out the truth of his initial impression.

However, checking is then a matter of *reflective* curiosity, not of simple, naïve, first order curiosity. Redirection and restrained inquisitiveness are here the road to virtue.

I think that some subtle distinctions are required here. In KC, I emphasize that we must carefully distinguish between checking whether* p* is true and checking of one’s own belief that *p* whether it is true. The second requires some form of self-reflection about one’s own beliefs while the first does not. This also holds for checking whether certain alternatives are true. Checking whether there is a painted mule in the pen instead of a zebra is not an instance of reflective checking, according to KC, because the target proposition is not about one’s own beliefs. Consequently, the checking procedure does not involve any self-reflection. In contrast, checking whether I am not deceived in believing that there is a zebra in the pen involves reflective checking, since it is about one’s own mental states.

In the next step, Miščević turns his attention to skepticism: He suggests that skepticism is a problem of overdoing curiosity. Properly used, curiosity is a virtue, but it can turn into a vice (following Miščević’s Aristotelian picture) if it is not appropriately deployed. In his words:

[D]igging too deeply will turn you into a skeptic. I would put it as a problem of uncontrolled reflective curiosity, of overdone zetetic work. Guido rightly places the issues of skepticism within the context of self-reflection. Here the skeptically minded inquirer digs too deeply and at a wrong place, thus producing a catastrophe. This brings us to the book’s central and concluding chapter which tackles the question that arises in discussing skepticism and bootstrapping: in what sense does the wish to check go too far, thus blocking knowledge? (Miščević, forthcoming)

Accordingly, Miščević (forthcoming) presents the following solution to this version of the skeptical problem:

Therefore, the subject should activate her self-trust, restrain her zetetic, inquisitive curiosity, and thus avoid the skeptical threat. Checking contrasts with ordinary self-reflection, which does not go as far in reflective curiosity as checking does.

Miščević connects this picture of viciously overdone curiosity with checking, suggesting that too much checking can turn us into a skeptic. Take the following quotes from Miščević (forthcoming):

In short, skepticism seems tied to excessive and wrong-headed inquisitiveness, perhaps it is even its result. Too intense checking can make irrelevant alternatives come into play, thus making them relevant. “Can I be sure this is not a painted mule?” is the crucial reflective question. Once it is raised, the desire to check arises, and it can easily go too far and turn into strong and active mistrust; it thus becomes an epistemic vice.

In the context of skepticism and bootstrapping, I distinguish checking whether one’s beliefs are true from ordinary self-reflection, which is a form of reflectively believing that one’s own beliefs are true that does not result from raising a question whether one’s own beliefs are true and intentionally using a method for settling it. Miščević suggests that ordinary self-reflection results from deploying curiosity in a virtuous way, whereas checking whether one’s own beliefs are true is vicious. In Miščević’s (forthcoming) words:

This will then be taken as pointing to the restraint solution. In short for Guido, ordinary self-reflection is not problematic, while checking one’s own beliefs is. To put it in virtue-epistemological terms, the ordinary self-reflection can go along with a virtue, while checking one’s own beliefs points to a vice. [...]

This will be understood as pointing to the restraint solution. In short, for Guido, ordinary self-reflection is not problematic, while checking one’s own beliefs is. To put it in virtue-epistemological terms, ordinary self-reflection can go along with a virtue, while checking one’s own beliefs points to a vice. [...]

How far may we go accepting epistemic offers from our senses, intuition, other people’s testimony, and so on? Guido does not name the stance required for knowledge. I would call the requisite quality scrupulosity. It goes with vigilance and investigative interest (curiosity), and it is closely connected with a desire to check but does not overdo it. Thus, my guess is that this is the virtue in the middle.

Let me connect Miščević’s picture of skepticism and curiosity with the central theses of KC. I do not have any objections to Miščević’s general comments on curiosity and skepticism. I think he is on the right track, and his theory on curiosity provides a highly valuable contribution to epistemology. However, I have some reservations to generalizing the view to checking. In cases of skepticism, too much curiosity can preclude us from knowing. Does too much *checking* also preclude us from knowing? This depends. If checking always involves suspension of judgment, then it does. However, checking does not generally require suspension of judgment—it only requires raising a question and intentionally using a method for settling it. Moreover, I doubt that, with skepticism, real suspension of judgment is involved. I do not think that we actually suspend judgment about whether we are brains in vats when reflecting on skepticism. Rather, checking in these contexts is always based on purely hypothetical checking. Thus, trying to check whether skeptical hypotheses are false, which we cannot successfully carry out as I argue in KC, does not preclude us from knowing.

Miščević (forthcoming) continues that “our drive to check motivates us to do the bootstrapping which then leads to epistemic defeat which results in the skeptic winning.” Perhaps there is a drive to check in some sense, though I would not say that we generally have a drive to check whether skeptical hypotheses are false. That is a project for very specific philosophical contexts. Moreover, I am unsure whether cases of checking whether the skeptical hypotheses are false typically lead us into bootstrapping. As I argue in KC, obvious bootstrapping is clearly a defective method for checking, so I think that we tend to refrain from attempting to check via bootstrapping. Since other potential methods, such as abductive reasoning, are also defective for checking, we remain with the correct intuition that we cannot check whether skeptical hypotheses are false.

To conclude, Miščević correctly notes that KC does not say much about motivations for checking and whether there can be virtuous and vicious motivations. This is a topic for further work on checking. Miščević (2020) provides an illuminating theory of curiosity as virtue and vice that provides a fruitful basis for such an extension of KC’s checking account. Miščević (forthcoming) summarizes his take on KC as follows:

In this paper I express my agreement with the central line of the book, culminating in the restraint solution of the skeptical puzzle, and neither question nor criticize it. Instead, I propose a virtue-epistemological interpretation of the restraint solution and re-interpret the problem of excessive checking as the problem of unbounded reflective curiosity.

I find Miščević’s theory on curiosity intriguing and, in my replies, I have presented some suggestions on how to connect it to checking.

## Robert Weston Siscoe: Checking and the Argument from Inquiry

It is a great pleasure to respond to Wes Siscoe’s thoughts, a very gifted and hardworking philosopher and a rising star of his generation. Siscoe has given me tremendous support by commenting on the whole manuscript of KC. The core claim that I make in KC about the intuitive connection between knowing and checking is captured by principle KSAC.

KSAC

In contexts of checking, when we raise the question whether *p* (or an alternative *q*) is true and deliberate about methods for settling this question, we tend to think that we do not know that *p* via strongly insensitive methods.

Siscoe thinks that there is a lacuna left, since KSAC makes a claim about knowledge intuitions but does not contain any explanation of why we should think in contexts of checking that knowledge requires a method that is not strongly insensitive. Siscoe (forthcoming) raises the following challenge for the view defended in KC:

One outstanding question for Melchior’s account is why, when we are in a checking context, we think that we do not know. This is one of the central claims of Knowing and Checking –without it, Melchior cannot explain why the sensitivity of checking would have any consequences for knowledge. Surprisingly, Melchior has very little to say about why KSAC is true. How could it be that, even though sensitivity is not necessary for knowledge, “we think in these contexts that knowing that* p* requires checking that *p*?”

Consequently, Siscoe addresses the question of why we believe that we lack knowledge when we are checking. He suggests that we can find the answer when considering the more general relation between knowing and checking. Here is Siscoe’s (forthcoming) project in his own words:

Checking is a form of inquiry, and many have argued that knowing and inquiring are incompatible, raising the possibility that KSAC can be supported by recent literature on the nature of inquiry.

Siscoe superbly overviews the current literature on knowledge and inquiry, pointing out that much discussion centers on the following ignorance norm:

Ignorance Norm (IN)

If one knows that *p*, then one ought not inquire into *p.*

In the remaining paper, Siscoe discusses the potential of IN for explaining or supporting the intuition that we do not know in checking contexts.

What would a connection between KSAC and IN look like? Let me reconstruct Siscoe’s suggestion as I understand it. First of all, as Siscoe correctly notes, KSAC provides an explanation of our intuitions about knowledge in checking contexts; KSAC is not a claim about whether these intuitions are *correct*. Accordingly, we need not to commit ourselves to a judgment as to whether IN is true or false for using it as an explanation of KSAC. Rather, we have to establish a connection between KSAC as a claim about our intuitions on knowing and checking and our *beliefs* in IN, which is a belief in the connection between knowledge and inquiry. If we believe IN, then we believe in contexts of inquiry that we do not know. Since contexts of checking are contexts of inquiry (and since we believe that), we also believe in contexts of checking that we do not know. Thereby, IN, or more precisely our beliefs in IN, provides an explanation of KSAC. I assume that something along these lines is the argumentation that Siscoe has in mind.

Let me address two aspects of Siscoe’s intriguing suggestion—first, the general connection between checking and inquiry, and second, the explanatory capacities of IN for KSAC. Siscoe claims that checking is a form of inquiry and he correctly does so. In KC, I focus on checking and its relation to knowing, leaving an analysis of the connection to inquiry for further research. However, the question about the relation between checking and inquiry is central and needs to be addressed. In KC (30), I provide the following definition of checking:

Checking

S checks whether *p* is true via method M only if

(1) S uses M with the intention of determining whether *p* is true.

(2) M is an appropriate method with respect to *p*, i.e., a method that fulfills certain modal conditions, in particular sensitivity.

Like checking, inquiring also involves raising a question and intentionally using a method for settling it. This common feature is the reason why checking is a form of inquiry. Successful checking requires using a method with the appropriate modal profile, in particular sensitivity. Do all forms of inquiry require a sensitive method? Plausibly not. Sensitivity is not a necessary condition on knowledge. If successful inquiry were to require a sensitive method, then there would be various forms of insensitive knowledge, like knowledge based on statistical evidence, which we cannot acquire via inquiry, and this seems implausible. Thus, checking requires sensitivity but other forms of inquiry do not.

One might think that the requirement of sensitivity is the only mark of distinction between checking and other forms of inquiry, but I think it is not. In Chapter 4 of KC, I outlined several different forms of checking. Checking simpliciter whether* p* is true requires the intention to check whether* p* is true and a method that is sensitive with respect to *p*, i.e., a method that would not indicate that* p* is true if* p* were false. Contrastive checking is checking whether* p* and not a particular alternative* q* is true. It requires fulfillment of a specific sensitivity condition, namely using a method that would not indicate that* q* is true if* p* were, and, importantly, the specific intention of the checking subject to check that* p* and not* q* is true. Hence, different forms of checking can be distinguished via the specific intentions of the checking subject, and the sensitivity of the method used. I think this distinction also holds for checking and inquiry. Checking not only involves the intention of determining whether* p* is true, as I analyze in KC, but the more specific *intention of checking* whether* p* is true. Other forms of inquiry plausibly involve other intentions. Thus, there is a difference on the purely subjective level between subjects who intend to check whether a proposition is true and a subject who aims to simply settle the question whether* p* is true. Moreover, checking and inquiry also differ concerning externalist features. Checking is associated with a higher epistemic standard than inquiry simpliciter, namely, fulfillment of sensitivity. Accordingly, an inquiring subject might correctly use insensitive methods such as statistical evidence for settling the question whether* p* is true, whereas a checking subject intuitively refrains from using insensitive methods. Thus, checking and other forms of inquiry diverge via two parameters. First, there is a difference between the intention of checking and the more general intention of settling a question, and second, checking requires the use of a sensitive method whereas other forms of inquiry do not.

Let me next investigate the explanatory relationship between KSAC and IN. KSAC, spelled out in detail, provides a twofold explanation:

Explanation 1: Conditions for entering a checking context

(C1) S_1_ raises the question whether *p* is true and deliberates about methods for settling this question and makes a knowledge judgment about herself.

(C2) S_1_ raises the question whether *p* is true and deliberates about methods for settling this question and makes a knowledge judgment about S_2_.

(C3) S_1_ believes that S_2_ raises the question whether *p* is true and that S_2_ deliberates about methods for settling this question and makes a knowledge judgment about S_2_.

Explanation 2: The standards for knowledge

In checking contexts, we tend to think that methods that are not checking methods are epistemically defective and cannot yield knowledge. We especially regard methods as defective that are strongly insensitive, i.e., random, opposing, or monotonous. (KC, 146)

The explanandum in question is our *intuition* that we do not know in contexts of checking. I start from the assumption that we think, correctly, that checking requires sensitivity. In contexts of checking, we regard the epistemic requirements for checking as crucial, which exclude the usage of a method that is strongly insensitive. We then generalize in checking contexts that strong insensitivity must be generally epistemically defective and therefore also inadequate for knowing. Thereby, the standards for checking are (correctly or not) transferred to knowledge. Therefore, we judge in checking contexts that we do not know via strongly insensitive methods. This is the line of argumentation pursued in KC.

Siscoe suggests that IN can provide explanatory support for KSAC. IN states that if one knows that *p*, then one ought not inquire into *p*. The crucial version of IN is one about our intuitions about knowledge, i.e., that we believe that IN is true (regardless of whether this belief is true.) IN can clearly not support KSAC in the sense that it can be a substantial premise for an argument for KSAC, since IN does not refer to any sensitivity condition on which KSAC centers. KSAC claims that we do not know via particular methods in contexts of checking. If we use IN an analogous way, then IN should, more generally, explain why we do not know in contexts of inquiry and therefore in contexts of checking simpliciter. In this sense, IN could support KSAC while making a more general point. However, I doubt that IN can accomplish this task. IN expresses a connection between knowledge and inquiry, namely, that in terms of knowledge we can formulate reasons for why we should or should not inquire, e.g., because in cases of knowledge the goal of inquiry is already reached. In this sense, knowledge explains why we should not inquire. Likewise, not knowing explains the permissibility of inquiry. However, the opposite explanatory relation seems flawed. The fact that we should not inquire does not structurally explain why we know. Likewise, the fact that it is permissible to inquire does not explain why we do not know. In fact, in virtue of knowing, we should not inquire and in virtue of not knowing, it is permissible to inquire. It is not the opposite way round that in virtue of inquiring we do not know and in virtue of not inquiring we know. Recall that KSAC is an explanation of why we think that we do not know in terms of (intuitions about) checking. Thus, this last order of explanation is in place when IN is used in analogy to KSAC, i.e., if IN were used to explain why one does not know in terms of the permissibility of inquiry. Such an explanation would get the metaphysical relation between knowledge and inquiry wrong. Therefore, I think that IN cannot structurally *explain* KSAC.

However, we must distinguish explanation from argumentation. Thus, the explanatory order does not preclude one from arguing that one inquires into whether* p* is true and, therefore, one does not know. However, this argumentation does not express the underlying structure of knowledge and inquiry. Analogously, one can abductively argue that the street is wet, and therefore, it had been raining without this argument expressing the underlying causal structure. Thus, we can use IN for arguing that (we believe that) if one inquires then, one does not know, but this kind of argument is not explanatorily illuminating. In contrast, KSAC does provide an explanation of why we think that we do now know in checking contexts via insensitive methods. Therefore, IN might provide some argumentative *support* for KSAC, as Siscoe suggests, but not a full explanation.

## Danilo Šuster: A Note on Knowing and Checking

Danilo Šuster is one of the most careful thinkers about epistemology, in particular about modal epistemology, that I know and I enjoy the intellectual exchange with him since many years. In his comments, Šuster (forthcoming) reflects on three points. First, he addresses the connection between sensitivity and safety by criticizing the claim defended in KC that there can be methods that are sensitive but unsafe. Second, he proposes to replace sensitivity with restricted sensitivity which only considers nearby possible worlds where the target proposition is false. Finally, Šuster reflects on my proposal to apply modal epistemology to logical necessities by considering impossible worlds. Let me address each of these points.

### Sensitive but Unsafe Methods

Orthodox modal epistemology as advanced by Nozick (1981), Sosa (1999), and Pritchard (2005) focuses on modal conditions on beliefs. Orthodoxy has it further that there are safe beliefs that fail to be sensitive, in particular beliefs in deception hypotheses. However, there are also cases proposed in the literature that aim at showing that there can be sensitive beliefs that fail to be safe.^19^ Orthodox modal epistemology focuses on the modal features of beliefs. My checking account, in contrast, focuses on the modal profile of methods. I am not convinced by the arguments for sensitive but unsafe *beliefs* discussed in the literature, in particular because they often do not take the belief forming method into account, but I argue in KC that there can be *methods* that are (weakly) sensitive but fail to be (weakly) safe. In KC (45), I present the following case:

The Stony Oracle

Suppose there is a stony oracle. If one consults the stony oracle in order to determine whether* p* is true, then a kind of miracle must happen so that the stony oracle makes any indications at all concerning *p*. Thus, in the nearest possible worlds where* p* is false and where the stony oracle is consulted to determine whether* p* is true, the stony oracle does not make any indication at all (and therefore does not indicate that* p* is true). Thus, asking the stony oracle is a weakly sensitive method with respect to *p*. Suppose further that there are some very remote worlds where the stony oracle indicates that *p*, and, in many of these worlds, it makes false indications that* p* is true. Therefore, asking the stony oracle is not a weakly safe method.

In this example, consulting Stony Oracle is a weakly sensitive but not weakly safe method because the nearest ~ *p* worlds which determine sensitivity are closer than the nearest worlds where the stony oracle indicates that *p*, which determine safety.

Šuster argues that the example of Stony Oracle, as formulated, fails, because in order to determine safety, we consider a neighborhood of nearby possible worlds, where S believes that* p* (or where method M indicates that *p*) but not very remote possible worlds as Stony Oracle requires. Our beliefs in an anti-skeptical hypothesis ~ sh is safe because there are no nearby possible worlds where ~ sh is false. According to Šuster, a belief that* p* formed via consulting Stony Oracle is safe because there are no nearby possible worlds where the Stony Oracle indicates that *p*. It is true that in the nearest possible worlds where S believes that* p* via method M,* p* is true. In both cases, it holds that safety is trivially fulfilled, albeit for different reasons. With skeptical hypotheses, there is no nearby possible world where* p* is true, and with the Stony Oracle, there is no nearby possible world where the method indicates that *p*.

Šuster is correct in that there are weaknesses in the Stony Oracle example that go unaddressed in KC. Nevertheless, I do not see why the example cannot be fixed. Safety accounts do not tell us much about the extent of the neighborhood of possible worlds that we have to consider. What we know is that worlds where we are brains in vats are too remote to be considered. What do we know about the extent of the neighborhood of possible worlds crucial for sensitivity? On the one hand, we can consult very remote possible worlds for determining sensitivity. On the other hand, there is also a minimal neighborhood of possible worlds that we always have to consider for determining sensitivity, a much-neglected fact in the literature. Call this neighborhood N1. Nozick judges that Barney’s belief in fake barn country that there is a barn in front of him is insensitive. Given this verdict, N1 must at least be so large to contain possible worlds where Barney is standing in front of a fake barn in fake barn country.

Suppose that the actual world is a ~ *p* world and so are all possible worlds in N1. If in N1, M does not indicate that *p*, then M is weakly sensitive with respect to *p*. If safety allows us to consider a modal neighborhood N2, which is larger than N1 but narrower than BIV-worlds, and in the area outside N1 but inside N2, there are many possible worlds where M indicates that* p* although* p* is false, then M is not weakly safe. Whether the case of Stony Oracle can be fixed depends on whether safety allows us to consider a larger space of possible worlds than the minimal neighborhood for sensitivity. If so, then there can still be sensitive but unsafe methods. While I agree with Šuster that there is a tension in the original formulation of Stony Oracle, I also do not see a principal reason that it cannot be fixed. To the best of my knowledge, there is no account about the neighborhood of worlds required for safety that rules out this possibility. Anyway, for the overall account of KC, not too much hinges on these issues since I assume that weak sensitivity as well as weak safety are necessary conditions for appropriate checking methods.

### Restricted Sensitivity

For determining safety, we consider a fixed neighborhood of possible worlds; however, this fixed neighborhood might exactly be specified. The possible world that we consider for sensitivity is context sensitive and includes the closest possible worlds where the target proposition is false, regardless of how remote those possible worlds might be. Šuster criticizes this difference between safety and sensitivity, suggesting that modal accounts in general should specify modally close, serious alternatives which must be considered. Consequently, sensitivity should be restricted to a specific neighborhood of possible worlds, a view that Šuster calls neighborhood reliabilism:

My hypothesis is that *N* corresponds to the sphere of seriously possible worlds, though this would imply that we might have to work with a more lax and contextually dependent notion of “remoteness.” Both safety and sensitivity should then be relativized to N, the sphere of seriously possible worlds. S’s true belief is safe just in case it turns out to be true whenever it is held in N. And S’s true belief is sensitive just in case in the closest possible worlds within N in which* p* is false, S does not believe that *p*. Nozickian sensitivity requires that we always consider at least one world where the actually true proposition is false, never mind whether this proposition is within N or not. But according to neighborhood reliabilism sensitivity is restricted, only potential error worlds within N are relevant for our knowledge assessments. (Šuster, forthcoming)

Šuster does not care too much about whether restricted sensitivity is still sensitivity (properly understood) but claims that it is the crucial modal condition. He argues that under these circumstances, restricted sensitivity becomes equivalent with safety, and I do not see any reason why he is not right about that.

Šuster then applies this concept of restricted sensitivity to examples of KC and, indeed, restricted sensitivity delivers different verdicts in some cases than unrestricted sensitivity. In KC, I discuss a case where a doctor consults only statistical evidence to determine whether a patient suffers from exotitis, a rare disease from which the patient only suffers in remote possible worlds. Because of the modal profile of exotitis, only consulting statistical evidence is a safe method. However, the method fails to be sensitive because, in the nearest possible worlds where the patient suffers from exotitis, consulting the statistical evidence alone indicates that she is not suffering from exotitis. However, if we consider restricted sensitivity instead of unrestricted sensitivity, then those worlds where the patient is suffering from exotitis are too remote to be considered. Thus, consulting the statistical evidence fulfills the condition of restricted sensitivity.

Let me reflect on Šuster’s intriguing proposal. First of all, if restricted sensitivity is equivalent with safety, then his proposal to replace sensitivity by restricted sensitivity amounts to eliminating sensitivity for safety. I do not claim that unrestricted sensitivity is always the crucial modal condition for theories in epistemology. In this respect, Šuster’s suggestion might be valuable in various contexts. However, I claim that unrestricted sensitivity, and not restricted sensitivity is crucial *for checking*. Šuster argues that we should only be concerned with serious alternatives. Whether an alternative is serious is either context-dependent or not context-dependent. This also holds for the case of exotitis. If it is not context-dependent, then having exotitis is not a serious alternative in any context, and thus, any completely arbitrary method like tossing a coin could count as a method for checking necessities. This seems implausible. Thus, being a serious alternative is context-dependent. Is suffering from exotitis then a serious alternative in the context of checking whether someone is suffering from exotitis? An answer to the negative seems implausible, since suffering from exotitis is the proposition in question in this particular context. Thus, in the context of checking whether* p* is true, *p is* a serious alternative that has to be ruled regardless of how modally remote it might be. However, ruling out remote but serious alternatives can only be guaranteed by unrestricted sensitivity, not by restricted sensitivity.

Šuster also defends the verdict that we can check in cases like exotitis by introducing a more complex picture which not only involves mere statistical evidence but more subtle Bayesian reasoning that fulfills restricted but not unrestricted sensitivity. He suggests that the appropriateness of checking methods can come in degrees and that the fact that methods are strongly insensitive in an unrestricted sense rules out methods like Bayesian reasoning in principle as proper for checking, an implausible consequence. Šuster (forthcoming) characterizes the situation between the checking doctor, D, and family members of the patient, as follows:

Bayesian reasoning (B) can be described as an action that a subject performs with a specific intention and the method used is responsive (in a certain broad sense) to the world (all empiricists since Hume would agree on that). The proper reply of P’s family members should not be: But you did not check (since your method is not weakly sensitive)! D can justifiably reply – I did, I made the calculations based on observation, statistical and inductive evidence. The family members would probably retort: this is not sufficient, you did not check *well* enough. A discussion might then ensue about what kind of checking is *appropriate* for the case at issue. If checking whether* p* is true implies using an appropriate method with respect to *p*, then I think that what counts as appropriate allows for degrees.

I agree with Šuster that Bayesian reasoning is a more appropriate and realistic method than simply consulting statistics, and I also agree that this method is, to a certain extent, responsive to the world. However, I do not share Šuster’s analysis of the case.

Šuster suggests that the controversy between the doctor and the family members is about whether D checked well enough, since methods can be more or less appropriate for checking. I do not object to the second observation about the gradual appropriateness of checking methods as long as the methods are not strongly insensitive, but I disagree about the subject of the dispute in the particular case. The dispute is not about whether D checked well enough, but about which proposition D checked. A far more reasonable objection against D’s claim to have checked is not that D did not check well enough, but that she checked something else, a different proposition, namely, how likely it is that the patient suffers from exotitis. Thus, the family members should reasonably reply: You did not check that the patient is not suffering from exotitis, you only checked what the likelihood is that she is suffering from exotitis (with the outcome that it is unlikely that she is suffering from exotitis). However, checking the likelihood of* p* does not entail checking whether* p* is true. In this respect, Bayesian reasoning is an inappropriate method for checking, as is merely consulting the statistics, and sensitivity remains a necessary condition on checking.

### Logical Necessities

Logical necessities are a notoriously difficult terrain for modal knowledge accounts. Any belief in a logical necessity is vacuously sensitive and safe, according to the standard Lewis-Stalnaker semantics for counterfactuals, and therefore trivially constitutes knowledge according to modal knowledge accounts, an undesired consequence. The checking account of KC is also affected by this problem, because every method, even tossing a coin, is sensitive and safe concerning logical necessities and therefore counts as a checking method if it delivers the correct result. The standard move for solving this problem for safety, as defended by Pritchard (2009) and Blome-Tillmann (2017), is to extend the method, not only taking the particular target proposition but also propositions in the neighborhood into account. Thus, an intuitively defective method that delivers the correct result for a logical necessity *n* is unsafe because it could easily deliver false results concerning propositions in the neighborhood of *n*. A similar line of argumentation is available for sensitivity. I challenge the standard view by presenting the following case:

René the Fermatist

Suppose that René lives in 1950 and is member of a cult called the Fermatists whose members believe all mathematical theorems that Pierre de Fermat ever proved plus his last theorem. They believe them based on a historical document that just lists these theorems but does not contain any proofs. René believes Fermat’s last theorem based on the document, a theorem that has not been proven by 1950. Moreover, all the other propositions that René believes via the document are also necessities. Thus, there is no nearby possible world where René uses the same belief forming method as in the actual world of consulting the document and where the resulting belief (including beliefs of other propositions) is false. Thus, René knows Fermat’s last theorem according to Pritchard’s revised account. (Melchior, 2021b, 719)

I regard the conclusion that René knows, as implausible, and therefore suggest rejecting Pritchard’s (2009) approach. In KC, I sketch an alternative route, considering not only possible but also impossible worlds for determining whether a method is sensitive or safe.^20^ According to this account, S’s belief that* p* is sensitive if, in the nearest possible or impossible worlds where* p* is false, S does not believe that *p*, and S’s belief is safe, if in the nearest possible or impossible worlds where S believes that *p*,* p* is true.

Šuster criticizes the impossible world accounts of KC, first by raising doubts about whether the impossibilism of KC delivers the correct result about Rene the Fermatist. Šuster objects that, given Fermat’s mathematical geniality, he would not have believed and written down his last theorem if it had been false. Thus, consulting the historical document is also sensitive according to impossibilism. Second, Šuster criticizes the impossible world account of KC more generally by raising general doubts about impossible worlds.

First, I do not find Šuster’s interpretation of the case very persuasive. There are good reasons to assume that Fermat did not have the mathematical tools at hand for proving that his last theorem is true. So why should not he believe that the theorem is true even it was false? Moreover, even if Šuster’s interpretation is correct, it seems that the example could easily be modified in order to meet this objection. Second, I am aware that impossible world semantics for counterfactuals are controversial, and I agree that, all else being equal, we should opt for the orthodox Lewis-Stalnaker semantics. However, I want to emphasize that the dispute is not primarily about choosing between an impossible world account and an account that is in line with orthodox semantics. There is an acknowledged agreement between defenders of orthodoxy, like Williamson (2017), and adherents of impossible world accounts that, intuitively, some counterpossibles (counterfactuals with impossible antecedents) are true and some are false. Impossible world accounts can do justice to this intuition, while the orthodox Lewis-Stalnaker semantics cannot since it predicts that all counterpossibles are true. Thus, orthodoxy must explain away our initial intuitions as false. One such explanation, spelled out by Williamson (2017), is that we have flawed heuristics which lead to the judgment that not all counterpossibles are true. However, I think that his explanation is profoundly and convincingly criticized by Berto Francesco et al. (2018) who provide a battery of objections against Williamson. Regardless of whether one shares this evaluation, the dialectical situation concerning logical necessities is not such that impossibilism has to motivate the introduction of impossible worlds in the first place. This would be an unfortunate stand point for impossibilism, given the challenges of impossible world accounts and the methodological advantages of Lewis-Stalnaker semantics. Rather the dialectical starting point is that orthodoxy has to motivate a possible world analysis of counterpossibles, given our opposing intuitions, and I do not think that has been convincingly achieved so far. Since impossible world accounts are in line with our intuitions about counterpossibles and no reason has emerged to explain away this impossibilist intuitions, I think that an impossibilist account for sensitivity and safety is on the right track.

## Footnotes

1 Hence, I do not hold, as Baumann (forthcoming, fn 25) claims, that knowledge is closed. In particular, I discuss in Chapter 8 of KC the often neglected contextualist option of intermediate contexts where knowledge-closure does not hold.

2 For a sensitivity-based knowledge account that also incorporates insensitive knowledge via deduction from sensitive knowledge, see Roush (2005; 2012).

3 For a specific generality problem for sensitivity, see Melchior (2014a); for a heterogeneity problem concerning skeptical hypotheses, see Melchior (2014b); and for problems concerning the heterogeneity of induction, see Melchior (forthcoming).

4 Baumann refers here to the plausible principle of counter-closure as discussed by Luzzi (2019).

5 For a further criticism of my solution to the generality problem, see Baumann (forthcoming), and for my reply, see the section on Baumann in this paper.

6 Becker also runs through various conflicting intuitions concerning sensitivity and induction. He is right that induction is a challenging field for sensitivity accounts, and backtracking counterfactuals do not provide a clearer picture. For a detailed account, see Melchior (forthcoming).

7 I provide the following analogy (KC, 78): Suppose that Barney is a rookie in a team of tax inspectors who are counting the barns of each color in the area for some specific tax purposes. It seems perfectly fine if a colleague says about Barney: ‘Barney does not know enough about barns in this area. So, we let him check the red barns and more experienced colleagues check barns in generals.’ Take the analogous example: Barney is a rookie member of a special unit that detects forged banknotes. There are very clumsy forged $100 bills circulating, but all other forged banknotes are very subtle. It is reasonable to say that Barney can check the authenticity of $100 bills but he cannot check the authenticity of bills in general. Becker criticizes the analogy that I draw by noting: The problem with the analogy is that there aren’t any clumsy red fake barns around. If there were, it would make sense to say that Barney can check for the real ones, since, being “clumsy”, the fakes would be easy to pick out, like the clumsily forged banknotes. If Barney checks that a particular bill is a $100 banknote, the whole point is that he’s checked that it’s a banknote—that’s his job—not just that it’s a reasonably-banknote looking thing with ‘$100’ written on it. This is very different than the red barn case, where Barney can’t discriminate any fakes. (If red barns could be faked, Barney wouldn’t be able to discriminate them, unlike forged $100 banknotes.). Becker is right on that point. The analogy is misleading. The case should be set up such that all $100 bills are real and, for all other bills, there are subtle copies circulating. In this case, it is reasonable to say that Barney can check whether (or how many) $100 bills are circulating but not whether (or how many) bills in general are circulating.

8 For influential discussions of bootstrapping, see Vogel (2000) and Cohen (2002).

9 See Murphy (2005).

10 For a discussion of these various moves, see Melchior (2015 and KC).

11 For applications in the context of epistemology, see Schaffer and Knobe (2012) and Gerken and Beebe (2016).

12 For contextualism, see Cohen (1988), DeRose (2009), and Lewis (1996); for subject-sensitive invariantism, see Hawthorne (2004), Fantl and McGrath (2009), and Stanley (2005).

13 See Melchior (2021a) for a sensitivity-based theory of discrimination.

14 David correctly points out that this formulation is misleading concerning the scope of the checking operator. It should be replaced by “Moreover, when we are not in a checking context, i.e., when we are not raising the question whether ~ *d* is true, we think that we know that ~ *d*.”.

15 One might object at this point that the argument could also be formulated for *being in a position to know* instead of knowing, which does not presuppose that the animal in the pen in a zebra, but I grant David’s point for the sake of the argument.

16 For a sensitivity-based theory of discrimination, see Melchior (2021a).

17 See Miščević (2020).

18 See Friedman (2019 and 2020) and Kelp (2021), among others.

19 See Alspector-Kelly (2011).

20 I develop this suggestion further in Melchior (2021b). For a discussion of logical necessities, see also Melchior (2017b).
